# The Prevalence of HIV-1 Drug Resistance among Antiretroviral Treatment Naïve Individuals in Mainland China: A Meta-Analysis

**DOI:** 10.1371/journal.pone.0110652

**Published:** 2014-10-24

**Authors:** Yingying Su, Fujie Zhang, Huixin Liu, M. Kumi Smith, Lin Zhu, Jing Wu, Ning Wang

**Affiliations:** 1 National Center for AIDS/STD Control and Prevention, Chinese Center for Disease Control and Prevention, Beijing, China; 2 Department of Epidemiology, University of North Carolina, Chapel Hill, North Carolina, United States of America; University of Rochester, United States of America

## Abstract

**Background:**

Surveillance of drug resistance in antiretroviral treatment-naïve patients in China is needed to ensure optimal treatment outcomes and control of the human immunodeficiency virus (HIV) epidemic.

**Methods:**

A systematic literature search was conducted in English and Chinese through PubMed (English), China National Knowledge Infrastructure (Chinese), Chinese Biomedical Literature Database (Chinese), and Wanfang (Chinese). Random effects models were used to calculate the pooled prevalence of transmitted drug resistance and subgroup analyses examined prevalence estimates across time periods, study locations, and study populations.

**Results:**

Analysis of data from 71 studies (47 in Chinese and 24 in English) yielded a pooled prevalence of transmitted HIV drug resistance to any antiretroviral drug class of 3.64% (95% confidence interval [CI]: 3.00%–4.32%). Rates were significantly high at initial stage of free ART program from 2003 to 2005 (5.18%, 95%CI: 3.13%–7.63%), and were much lower among studies conducted in 2006–2008 (3.02%, 95%CI: 2.03%–4.16%). A slight increase was observed again in the most recent study period from 2009 to 2012 (3.68%, 95%CI: 2.78%–4.69%). Subgroup analysis revealed highest prevalence levels of transmitted drug resistance in Beijing city, and Henan and Hubei provinces (above 5%), and although differences in prevalence rates among risk groups were negligible, men who have sex with men were unique in their relatively large portion of protease inhibitor resistance, a second-line drug of limited availability in China.

**Conclusions:**

Overall prevalence of transmitted HIV drug resistance in China is classified as “low” by the World Health Organization. However regional and temporal variability suggest a more complex epidemic for which closer HIV drug resistance surveillance is needed. A nationwide HIV drug resistance surveillance system to monitor both treatment-experienced and treatment-naïve patients will be a cornerstone to ensure the effectiveness of treatment scale-up, particularly as China seeks to expand a national policy of antiretroviral treatment as prevention.

## Introduction

China has one of the largest populations of persons infected with human immunodeficiency virus type-1(HIV-1). As of 2011 an estimated 780,000 people living with HIV/AIDS and among them 154,000 living with AIDS in China [1]. The HIV epidemic is largely concentrated in injecting drug users (IDU), as well as in groups with higher risk sexual exposures including female sex workers and men who have sex with men (MSM) [2]. As of 2007, however, data from the national case report system has suggested that heterosexual transmission (HST) has become the primary mode of transmission in China, signaling a potential transmission to a more generalized epidemic [1].Of the about 70,000 new HIV diagnoses from January to September in 2013, those believe to be acquired through unprotected sex accounted for 89.9% (heterosexual transmission for 69.1%, homosexual transmission for 20.8%) [3].

To combat the spread of HIV through the general population, the Chinese Center for Disease Control has launched a policy initiative to harness suppressive antiretroviral therapy (ART) to slow the spread of HIV in China. The existing national free ART program established in 2003, is expected to provide a strong foundation for such an effort. Initially started as a pilot program for patients who were former plasma donors (FPD), the program has undergone rapid scale up [4], [5]. And by the end of September 2013, the national free ART program has treated about 260,000 cumulative patients [3], and has reduced AIDS associated mortality from 39.3 to 14.2 deaths per 100 persons from 2002 to 2009 [6].

Rapid scale up of ART access has been accompanied by a concomitant rise in drug resistance among treated HIV patients. This has been largely attributed to the limited selection of antiviral agents through the national ART program on which most patients rely, and the lack of critical disease monitoring tools like CD4 or viral load testing in most treatment settings [5], [7], [8]. Though the relationship between poor treatment outcomes, insufficient viral suppression, and emerge of drug resistance is well understood [9], [10], little is known about onward transmission of resistant strains in Chinese settings. Primary infection with drug resistant strains severely limits new patients' treatment options and shortens time to treatment failure. The prevalence of transmitted drug resistance (TDR) in wider circulation could also undermine program efficacy of the national treatment efforts [11]. A better understanding of the prevalence of drug resistance in treatment naïve HIV/AIDS patients in China will provide insight critical for both clinical management and for broader disease control efforts [12].

The goal of this systematic review is to provide an overview and pooled prevalence estimate of HIV drug resistance among treatment-naïve patients in China. We also provide a simple analysis of several trends and distributions available from the data in order to provide more insight into better treatment and control of HIV in China.

## Methods

### Search strategy

We conducted a systematic literature in English and Chinese using the following four databases: PubMed, China National Knowledge Infrastructure (CNKI), Chinese Biomedical Literature Database (CBM), and Wanfang, from January 1, 1990 through February 28, 2014. Search terms included (“HIV” OR “AIDS” OR “human immunodeficiency virus” OR “acquired immunodeficiency syndrome”) AND (“drug resistance” or “drug resistant”) AND (“China”). Details on search strategy can be found in the Appendix S1. We further reviewed the reference lists for other relevant articles not captured in our initial search.

### Selection Criteria

Investigators YYS and HXL independently assessed eligibility of articles and performed the data extraction. Discrepancies between the two investigators were resolved by consulting the other authors (NW and MKS). Eligible articles all met the following inclusion criteria: (1) inclusion of data on HIV-1-infected and treatment-naïve individuals; (2) availability of basic demographic information for study subjects such as age, sex, or most likely HIV transmission route; (3) availability of information on study design, location, and period; and (4) HIV drug resistance patterns were interpreted using the WHO recommended Stanford HIV Drug Resistance Database (http://hivdb.stanford.edu). Articles that restricted study subjects to HIV patients of a single subtype were excluded, as were those without information on patient treatment status or that pooled results of treated and untreated patients. Studies based outside of mainland China such as Taiwan, Hong Kong and Macao were excluded due to the fact that these regions maintain autonomous and separate HIV/AIDS treatment programs. Articles based on the same or overlapping study populations were included only once to avoid duplication, with preference given to the article with more detailed information about the population, or, all else equal, with the larger sample size.

### Data extraction

A standardized form was used to assess article eligibility and to extract data including author names, year of publication, study period, language of article, study location, age range and sex of participants, most likely routes of primary HIV infection, time of infection(chronic or new diagnose), study design (cohort vs. cross section), recruitment method, system of HIV drug resistance typing, portion of tested samples whose HIV RNA was successfully amplified and sequenced, and characteristics of the sequences genotype including number of mutations with at least low-level resistance 1) to any antiretroviral agent, 2) to nucleoside reverse transcriptase inhibitor(NRTI), 3) to non-nucleoside reverse transcriptase inhibitor(NNRTI), 4) to protease inhibitor(PI).

### Statistical analysis

The proportion of TDR was defined as number of samples exhibiting at least a lower-level resistance to any antiretroviral agent, divided by the total number of samples successfully amplified and sequenced [13]. This definition of TDR prevalence is consistent with that used for the WHO TDR surveillance mutation list [14]. The Freeman-Tukey Double arcsine transformation of proportions was used to calculate an overall proportion. Model choice (fixed versus random effects models) was based on heterogeneity assessment by examining the Cochran *Q* statistic (p-value of <0.1 was considered a statistically significant) [15] and the *I^2^* statistic (values of 25%, 50% and 75% are considered to represent low, medium and high heterogeneity respectively) [16]. The DerSimonian-Laird estimate was used in case of random effects. Subgroup analyses were conducted according to study period, study location and study population to assess the heterogeneity between studies. Publication bias was assessed with the funnel plot and Egger's test, which found no evidence of bias (Figure S1). Analyses were carried out using R EpiTools package [17] and SAS(version 9.3, SAS Institute).

## Results

### Study selection and characteristics

**Figure 1:**
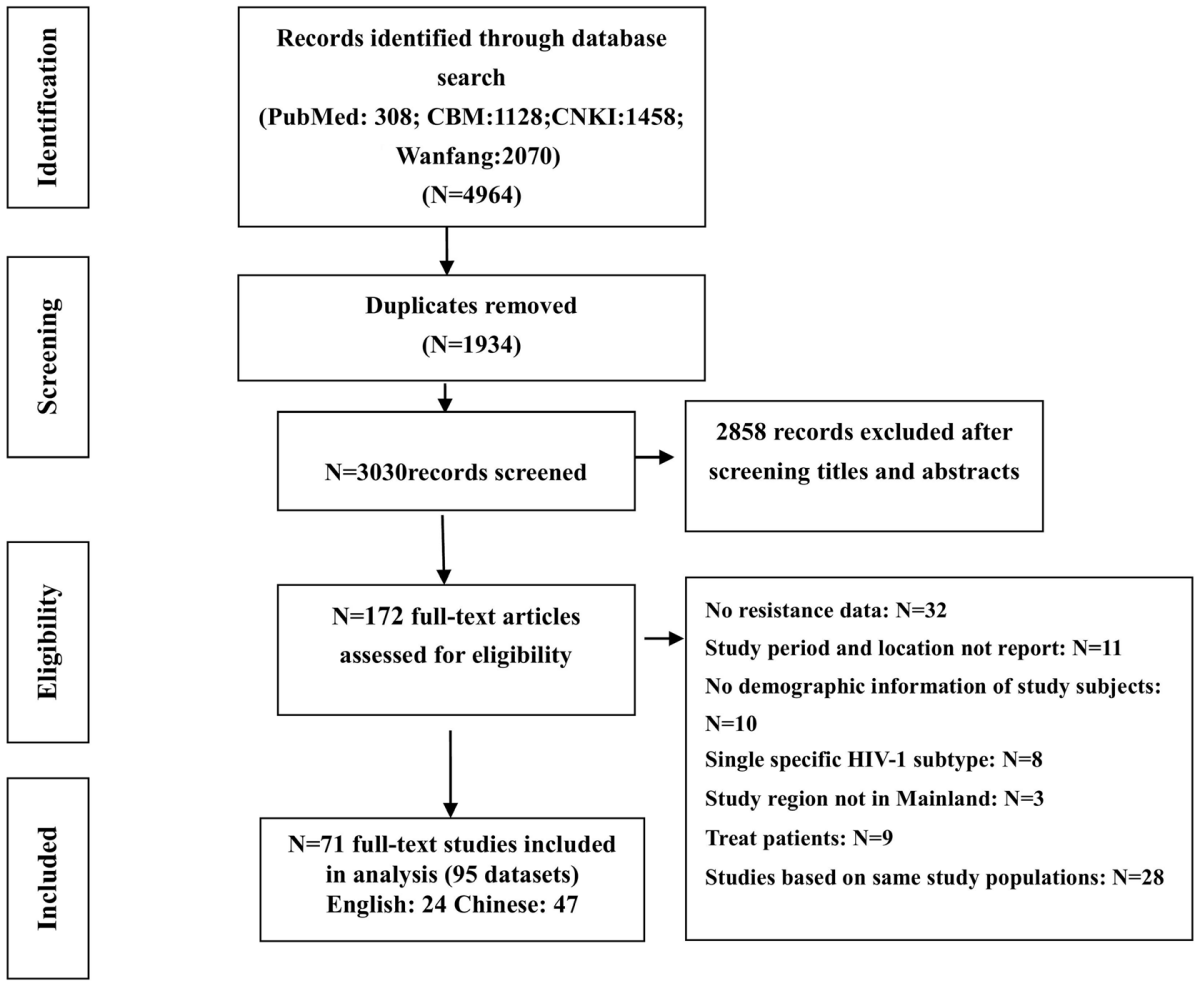
Flow chart of the process of studies selection.

Of the 4964 articles (4656 in Chinese, 308 in English) initially identified, of which 1934 were excluded as duplicates, another 2858 were removed for relevance based on a title and abstract screening. Following a closer full text review of the remaining 172 articles we identified 71 studies for inclusion in the final analysis, of which 47 were in Chinese, 24 in English (Figure 1). Seventeen of these employed the WHO HIV drug resistance survey method which recommends use of truncated sequential sampling (TSS) to classify levels of transmitted drug resistance for each drug class in resource poor settings [18]. Among the remainder, 54 were cross-sectional studies. Details on resistance to specific classes of antiretroviral drug were available in 68 studies (Table S1).

### Transmitted drug resistance prevalence rates

The 71 studies included in this analysis represented results from a collective total of 11,633 HIV-1 treatment naïve individuals, among whom 9,167 HIV RNA sequences were successfully amplified and analyzed. The random effects estimate for overall pooled prevalence of HIV-1 TDR to any antiretroviral drug was 3.64% (95% confidence interval [CI]: 3.00%–4.32%). The pooled prevalence of mutations resistant to NRTI's, NNRTI's, and PI's was 0.91% (95%CI: 0.60%–1.27%), 1.14% (95%CI: 0.75%–1.59%), 0.68% (95%CI: 0.41%–1.01%), respectively.

**Figure 2:**
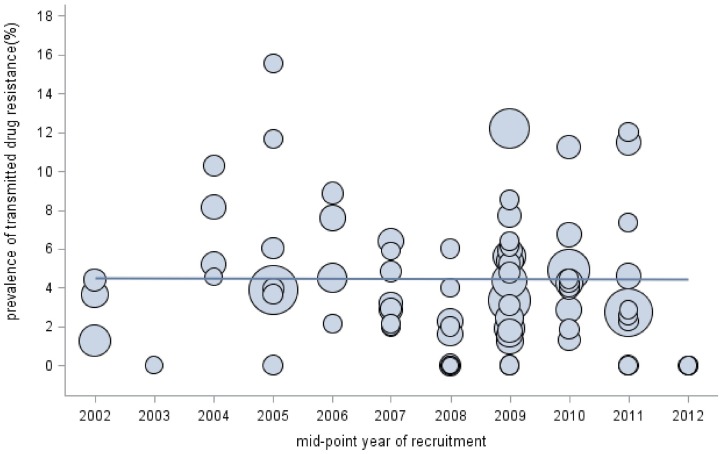
Temporal trends of prevalence of drug resistance in HIV ART-naïve patients in China. The size of circle correspond to sample size of every study, 81 estimates from 71 articles were used in this subgroup analysis.

**Figure 3:**
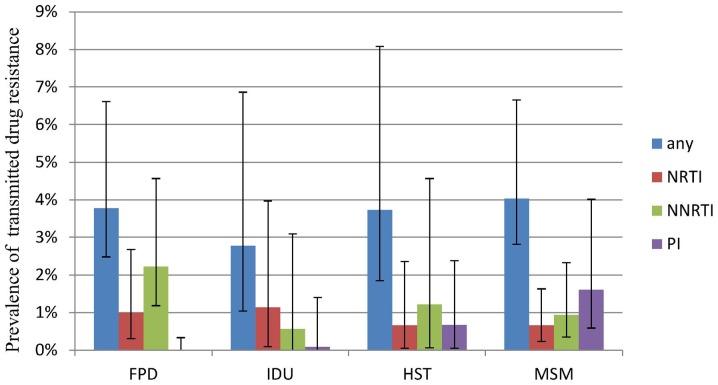
Prevalence of transmitted drug resistance in difference risk groups. Note: MSM: men who have sex with men; HST: Heterosexual transmission; FPD: former plasma donors; IDU: Injecting drug users; NRTI: nucleoside reverse transcriptase inhibitor; NNRTI: non-nucleoside reverse transcriptase inhibitor; PI: protease inhibitor.

TDR prevalence was further explored within identifiable subgroups in studies where relevant information was provided (Table 1). Figure 2 shows temporal trends in TDR prevalence according to the midpoint of the year in which study participants were recruited. A slightly upward trend can be detected in the composite time trend, though the trend was not statistically significant. We further categorized study times into four time periods roughly corresponding to the following phases of the national free ART program: pilot (before 2003), initiation (2003 to 2005), scale-up (2006 to 2008), and introduction of second line therapy (2009–2012) [5], [8]. Results of the analyses showed that the overall TDR prevalence for any antiretroviral drug class was significantly high at initial stage of free ART program (5.18%, 95%CI: 3.13%–7.63%), but much lower during the scale-up phase (3.02%, 95%CI: 2.03%–4.16%). TDR prevalence rose again slightly after 2008 (3.68%, 95%CI: 2.78%–4.69%). Geographic subgroup analyses excluded studies in which the study represented the sole report on TDR from a given province [19]–[23] or in which results were reported as pooled prevalence of patients from multiple provinces [24]–[32]. Stratified analyses showed that TDR prevalence rates in the city of Beijing and the provinces of Henan and Hubei were all above 5%, but the rest were below this threshold. Finally, where information on most likely routes of HIV infection was provided, we estimated TDR prevalence for the following groups: FPD (5 articles), MSM (17 articles), IDU (5 articles), and those otherwise described as heterosexual individuals (3 articles). Pooled prevalence of TDR in these four risk groups were highest among MSM (4.03%, 95%CI:2.81%–5.43%), and slightly lower among FPD (3.78%, 95% CI: 2.48%–5.31%) and heterosexuals (3.73%, 95% CI: 1.85%–6.20%). Rates were lowest among IDU (2.78%, 95% CI: 1.04%–5.12%), but substantial overlap of the 95% confidence intervals suggests no significant difference in TDR rates among these groups. Figure 3 shows the prevalence of TDR to specific classes of antiretroviral drug within each subgroup, in which a disproportionately large prevalence of PIs resistance can be seen in MSM.

**Table 1:** Result of stratified meta-analysis based on time period, study region and risk group.

Category	Number of estimates	Pooled prevalence (%)	95%CI (%)	Heterogeneity p-value^a^
Time period^b^				
before 2003	3	2.65	0.89–5.17	0.15
2003–2005	5	5.18	3.13–7.63	0.01
2006–2008	20	3.02	2.03–4.16	0.18
2009–2012	53	3.68	2.78–4.69	<0.0001
Region^c^				
Hubei	3	7.37	4.61–10.65	0.42
Henan	7	7.11	4.10–10.80	0.00
Beijing	5	6.52	4.35–9.05	0.27
Shanghai	3	4.91	2.25–8.38	0.87
Tianjin	2	4.90	1.25–10.31	0.65
Zhejiang	2	4.76	2.35–7.86	0.51
Liaoning	2	4.40	2.26–7.15	0.95
Guangdong	7	3.43	2.17–4.91	0.70
Guangxi	6	2.72	1.70–3.94	0.14
Hunan	2	2.69	0.91–5.22	0.83
Yunnan	11	2.44	1.14–4.11	0.08
Guizhou	2	2.25	0.00–14.08	0.04
Anhui	3	1.89	0.02–5.51	0.60
Shandong	2	1.56	0.07–4.24	0.67
Sichuan	3	0.49	0.00–2.16	0.24
Risk group^d^				
MSM	17	4.03	2.81–5.43	0.05
FPD	5	3.78	2.48–5.31	0.19
HST	3	3.73	1.85–6.20	0.05
IDU	5	2.78	1.04–5.12	0.22

Note: ^a^Heterogeneity p value was calculated by examining the Cochran *Q* statistic (p-value of <0.1 was considered a statistically significant); ^b^For time period subgroup analysis, 81 HIV TDR prevalence estimates from 71 articles were used; ^c^For study region subgroup analysis, 60 HIV TDR prevalence estimates from 58 articles were used; ^d^For risk group subgroup analysis, 30 HIV TDR prevalence estimates from 28 articles were used. MSM: men who have sex with men; FPD: former plasma donors; HST: Heterosexual transmission; IDU: Injecting drug users.

## Discussion

Our meta-analysis showed that estimated prevalence of TDR in ART-naïve patients in China was 3.64% (95%CI: 3.00%–4.32%). Such a pooled prevalence rate of primary drug resistance is similar to those from other developing country settings such as Thailand (0.5%, 95% CI: 0.1%–1.4%) and India (2.7%, 95% CI: 1.1%–4.7%) [33], but lower than those estimated in Europe (8.4%, 95%CI: 7.4%–9.5%) [34] and the United States (14.6%, 95% CI: 12.9%–16.0%) [35]. The far lower rates of TDR in Chinese HIV patients as compared to those in western countries may be due to differences in sampling strategies, but may also reflect real differences in the prevalence of circulating strains of drug resistance HIV in the two settings. China's HIV epidemic is at a relatively earlier phase compared to those in Africa and the west, as has a shorter history of population level exposure to ART, leading to less drug resistance in the population.

Changes in estimated TDR prevalence across populations sampled over distinct time periods likely reflect population level response to the antiretroviral drug used most widely by beneficiaries of China's free ART program at each phase. Higher TDR prevalence estimates in the earliest phase of the national free ART program may be due to the widespread use of generic agents including zidovudine, didanosine, and nevirapine [5]. Subsequent survey work as shown a strong association between didanosine use and virological failure in the earliest participants of the free ART program, suggesting high drug resistance rates due to antiretroviral drug choice [36], [37]. In addition, ART dispensation in the early phase of the epidemic relied on village clinics where clinicians who often lacked formal medical training and who worked without the benefit of routine laboratory monitoring to aid their patient management [5], [7], [38]. After 2005, routinization of ART practices [39] and availability of branded drugs including lamivudine and efavirenz may have led to lower population level prevalence of drug resistant strains [4]. Slightly higher rates of TDR following 2008 may be due to broad expansion of ART access during this phase [8], [40] or due to a cumulative increase in the prevalence of drug strains in HIV infected person on therapy [41]. However the lack of a statistically significant difference in pooled prevalence of TDR in these two periods may reflect a spurious difference.

Geographic sub-analyses showed that pooled estimates of TDR were highest in Beijing (a provincial-level city) and Henan and Hubei provinces. Higher rates of TDR in Beijing may be due to the fact that three out of the five studies based in Beijing were of MSM [42]–[44]. Past reports of TDR in MSM in Beijing have been high [42]—as compared to rates among MSM recruited in Guangzhou city [45], Liaoning province [46] and Yunnan province [47]—but for reasons that are still poorly understood. Higher rates in Henan province likely reflect the fact that it has the largest concentration of treated HIV patients in China with the earliest access to ART due to the piloting of the national treatment program in this population [4]. Finally, the three articles conducted in Hubei province were carried out in the early phase of the free ART program, a possible explanation for the higher prevalence of TDR.

Though drug resistance patterns did not vary significantly across risk populations, one noteworthy finding was the elevated prevalence of PI-specific TDR among MSM. The fact that PI's were not available through China's free ART program until 2008 [48] and its limited use even today [25] may suggest acquisition of PI-specific TDR from individuals treated outside of the free ART program or foreign nationals treated abroad. Mixing between foreign-born and Chinese men in MSM sexual networks, and the relative affluence of MSM (or the subset captured in epidemiological studies) which allows for increased international travel [25] may be partial explanations for this phenomenon.

Our analysis faces several important limitations. Convenience sampling was the most common recruitment method for most studies included in this analysis which limits representativeness of findings. Selection bias may also have led to oversampling of studies in regions with high levels of ART penetration such as Guangdong, Beijing, Yunnan, Henan and Guangxi.

Despite these limitations, findings from this study represent the first comprehensive investigation of HIV TDR in treatment naïve individuals in China. Though estimated rates are lower than many western settings, the relative infancy of the Chinese ART program underscores the importance of consistent monitoring of onward transmission of drug resistant HIV at the population level. Beyond the clinical considerations of limited therapeutic options for TDR in treatment naïve patients, China's focus on treatment as prevention as part of its newly articulated HIV control strategy in the 12th Five-Year Action Plan [12] underscores the HIV prevention tradeoffs of expanded ART access.

## Supporting Information

Figure S1 **Funnel plot.**
(DOC)

Table S1 **Characteristics of included studies.**
(DOC)

Checklist S1 **PRISMA checklist.**
(DOC)

Appendix S1 **Detailed search strategy.**
(DOCX)
