# Supplementary material for: The Prevalence of HIV-1 Drug Resistance among Antiretroviral Treatment Naïve Individuals in Mainland China: A Meta-Analysis
Source: PLoS One. 2014 Oct 24;9(10):e110652. doi: 10.1371/journal.pone.0110652 (PMC4208788; doi:10.1371/journal.pone.0110652)
Supplement: Figure S1 — Funnel plot. (DOC) [file pone.0110652.s001.doc]

**Figure S1. Funnel plot**

Egger’s test: test statistic= -1.3019, p-value = 0.1973
