# Supplementary material for: The Prevalence of HIV-1 Drug Resistance among Antiretroviral Treatment Naïve Individuals in Mainland China: A Meta-Analysis
Source: PLoS One. 2014 Oct 24;9(10):e110652. doi: 10.1371/journal.pone.0110652 (PMC4208788; doi:10.1371/journal.pone.0110652)
Supplement: Table S1 — Characteristics of included studies. (DOC) [file pone.0110652.s002.doc]

Table S1 Characteristics of included studies

| Study population | First author, published year | study period | study site | Sampling method | Sample Size | Samples successfully amplified and sequenced | Number of each type of mutation (N) | | | | | | |
| --- | --- | --- | --- | --- | --- | --- | --- | --- | --- | --- | --- | --- | --- |
|  |  |  |  |  |  |  | Any | NRTI | | NNRTI | | PI | |
| FPD | Guo WS(2006)[1] | 2005 | Henan | Random | 76 | 76 | 3 | | 1 | | 2 | | 0 |
|  | Tang H(2007)[2] | 2004-2005 | Hubei | convenience | 135 | 115 | 6 | | 1 | | 4 | | 1 |
|  | Cao XL(2010)[3] | 2007 | Henan | Random | 105 | 105 | 3 | | 1 | | 3 | | 0 |
| HST | Li L(2013)[4] | 2009 | Guangxi | Random | 253 | 211 | 4 | | 2 | | 1 | | 1 |
| IDU | Liao L(2007)[5] | 2003-2004 | Multiple provinces | convenience | 25 | 25 | 0 | | 0 | | 0 | | 0 |
|  | Yu GL(2009)[6] | 2007 | Guangdong | convenience | 63 | 49 | 1 | | 1 | | 0 | | 0 |
|  | Han XX (2012)[7] | 2009-2010 | Xinjiang | convenience | 77 | 77 | 1 | | 1 | | 0 | | 0 |
| MSM | Zhang X(2007)[8] | 2002-2006 | Beijing | convenience | 54 | 45 | 7 | | 3 | | 3 | | 1 |
|  | Zhao GL(2009)[9] | 2007-2008 | Guangdong | convenience | 100 | 94 | 3 | | 1 | | 2 | | 1 |
|  | Zheng MN(2010)[10] | 2008 | Tianjin | Random | 67 | 50 | 3 | | 1 | | 2 | | 0 |
|  | Yuan D(2011)[11] | 2007-2009 | Sichuan | convenience | 98 | 77 | 0 | | 0 | | 0 | | 0 |
|  | Wu YS(2011)[12] | 2007-2010 | Beijing | convenience | 202 | 149 | 8 | | 1 | | 2 | | 7 |
|  | Zhao B (2011)[13] | 2003-2009 | Liaoning | convenience | 217 | 201 | 9 | | 1 | | 1 | | 9 |
|  | Li L(2011)[14] | 2008-2009 | Hebei | Random | 21 | 15 | 0 | | 0 | | 0 | | 0 |
|  | Zhao GL(2012)[15] | 2008-2010 | Guangdong | convenience | 227 | 164 | 4 | | 2 | | 3 | | 0 |
|  | Lei YH(2012)[16] | 2011-2012 | Anhui | convenience | 35 | 35 | 1 | | 0 | | 1 | | 0 |
|  | Huang D(2013)[17] | 2010 | Guangdong | convenience | 144 | 141 | 4 | | 3 | | 1 | | 0 |
|  | Li L(2013)[18] | 2007-2010 | Beijing | Random | 95 | 76 | 4 | | 0 | | 0 | | 4 |
|  | Yang J (2013)[19] | 2010 | Nineteen provinces | convenience | 610 | 489 | 24 | | 5 | | 3 | | 19 |
|  | Bu P(2013)[20] | 2011 | Guizhou | convenience | 49 | 41 | 3 | | 0 | | 2 | | 1 |
|  | Chen M(2014)[21] | 2010-2012 | Yunnan | convenience | 131 | 131 | 6 | | 2 | | 3 | | 3 |
| Multiple risk groups | Liu L(2005)[22] | 2004 | Sichuan | Random | 41 | 22 | 1 | | 1 | | 1 | | 0 |
|  | Kuang JQ(2007)[23] | 1998-2006 | Twenty provinces | convenience | 326 | 237 | 3 | | 1 | | 1 | | 1 |
|  | Zhong P(2007)[24] | 2005 | Shanghai | Random | 60 | 55 | 2 | | 1 | | 0 | | 1 |
|  | Han X(2007)[25] | 1999-2004 | Liaoning | convenience | 91 | 91 | 4 | | 1 | | 0 | | 3 |
|  | Yao YP(2008)[26] | 2004-2006 | Zhejiang | convenience | 99 | 83 | 5 | | 2 | | 3 | | 1 |
|  | Chen X(2008)[27] | 2007 | Hunan | TSS | 79 | 69 | 2 | | 1 | | 1 | | 0 |
|  | Hei FX(2009)[28] | 2008 | Beijing | TSS | 61 | 50 | 2 | | 1 | | 0 | | 1 |
|  | Yuan Y(2009)[29] | 2007-2008 | Henan | TSS | 69 | 50 | 1 | | 0 | | 1 | | 0 |
|  | Luo M(2009)[30] | 2003-2005 | Hubei | convenience | 150 | 123 | 10 | | 4 | | 6 | | 1 |
|  | Lin B (2010)[31] | 2009 | Shandong | convenience | 168 | 128 | 2 | | 0 | | 1 | | 1 |
|  | Liao L(2010)[32] | 2004-2005 | Fourteen provinces | Random | 967 | 676 | 26 | | 11 | | 14 | | 3 |
|  | Zhang J (2010)[33] | 2006 | Shandong | TSS | 53 | 47 | 1 | | 0 | | 1 | | 0 |
|  | Liu L(2011)[34] | 2008-2009 | Shanghai | convenience | 118 | 118 | 7 | | 3 | | 4 | | 2 |
|  | Ma YL(2011)[35] | 2009 | Yunnan | TSS | 56 | 47 | 4 | | 1 | | 2 | | 1 |
|  | Wu JJ(2011)[36] | 2008 | Anhui | TSS | 58 | 29 | 0 | | 0 | | 0 | | 0 |
|  | Zhao J (2011)[37] | 2008-2010 | Guangdong | TSS | 238 | 144 | 5 | | 2 | | 0 | | 3 |
|  | Liang SJ(2011)[38] | 2007 | Guangxi | TSS | 70 | 47 | 1 | | 0 | | 0 | | 1 |
|  | Zhang JF(2012)[39] | 2009-2011 | Zhejiang | TSS | 182 | 165 | 7 | | 3 | | 1 | | 5 |
|  | Xue XJ(2012)[40] | 2011-2012 | Henan | convenience | 275 | 192 | 21 | | NA | | NA | | NA |
|  | Chen M (2012)[41] | 2011 | Yunnan | TSS | 62 | 44 | 1 | | 0 | | 1 | | 0 |
|  | Chen M(2012)[42] | 2011 | Yunnan | TSS | 62 | 54 | 1 | | 0 | | 0 | | 1 |
|  | Xue XJ(2012)[43] | 2007-2011 | Henan | convenience | 204 | 204 | 15 | | NA | | NA | | NA |
|  | Wu J(2012)[44] | 2010 | Shanghai | Random | 77 | 45 | 2 | | 1 | | 1 | | 0 |
|  | Yang HT(2012)[45] | 2009 | Jiangsu | TSS | 50 | 47 | 0 | | 0 | | 0 | | 0 |
|  | Bu P (2012)[46] | 2008-2009 | Guizhou | TSS | 47 | 47 | 0 | | 0 | | 0 | | 0 |
|  | Wang X (2012)[47] | 2010 | Tianjin | convenience | 79 | 51 | 2 | | 2 | | 0 | | 0 |
|  | Li WJ(2012)[48] | 2009-2012 | Heilongjiang | convenience | 39 | 39 | 1 | | 1 | | 0 | | 0 |
|  | Wang X(2012)[49] | 2011 | Five provinces | Random | 729 | 627 | 17 | | 5 | | 8 | | 4 |
|  | Chen M (2012)[50] | 2009-2010 | Yunnan | convenience | 320 | 299 | 13 | | 1 | | 8 | | 4 |
|  | Yang C(2012)[51] | 2009-2011 | Guangdong | convenience | 157 | 119 | 8 | | 5 | | 5 | | 0 |
|  | Ye JR(2012)[52] | 2006-2007 | Beijing | convenience | 200 | 145 | 11 | | 3 | | 5 | | 5 |
|  | Su Q(2012)[53] | 2008 | Guangxi | convenience | 144 | 124 | 2 | | 1 | | 0 | | 1 |
|  | Chen M (2013)[54] | 2011 | Yunnan | TSS | 59 | 41 | 0 | | 0 | | 0 | | 0 |
|  | Qiu LJ(2013)[55] | 2003-2010 | Fujian | convenience | 125 | 125 | 8 | | 6 | | 1 | | 2 |
|  | Li L(2013)[56] | 2009-2010 | Three provinces | convenience | 1746 | 1159 | 82 | | 51 | | 57 | | 13 |
|  | Zeng P(2013)[57] | 2007-2010 | Sichuan | convenience | 244 | 159 | 2 | | 0 | | 2 | | 0 |
|  | He JM(2013)[58] | 2009-2012 | Hunan | TSS | 182 | 182 | 5 | | 0 | | 1 | | 4 |
|  | Chen M(2013)[59] | 2012 | Yunnan | TSS | 68 | 45 | 0 | | 0 | | 0 | | 0 |
|  | Chen M(2013)[60] | 2012 | Yunnan | TSS | 50 | 36 | 0 | | 0 | | 0 | | 0 |
| no risk group defined | Si XF(2004)[61] | 2002 | Twenty-one provinces | Random | 164 | 164 | 6 | | 3 | | 2 | | 1 |
|  | He WH(2006)[62] | 2004 | Hubei | convenience | 84 | 68 | 7 | | NA | | NA | | NA |
|  | Li HP(2007)[63] | 2004-2005 | Guangxi | convenience | 58 | 43 | 5 | | 0 | | 2 | | 3 |
|  | Zeng P(2008)[64] | 2007-2010 | Five provinces | convenience | 172 | 113 | 2 | | 1 | | 1 | | 0 |
|  | Tu YQ(2009)[65] | 2006-2007 | Henan | convenience | 39 | 34 | 2 | | 0 | | 2 | | 0 |
|  | Bi ZY(2009)[66] | 2007-2008 | Guangxi | Random | 72 | 62 | 3 | | 0 | | 3 | | 1 |
|  | Tu YQ(2009)[67] | 2005-2006 | Yunnan | convenience | 52 | 47 | 0 | | 0 | | 0 | | 0 |
|  | Yin CY(2011)[68] | 2004-2008 | Four provinces | convenience | 88 | 79 | 7 | | 3 | | 4 | | 0 |
|  | Qian YC(2012)[69] | 2008-2011 | Anhui | convenience | 65 | 65 | 2 | | 1 | | 0 | | 1 |
|  | Chen S (2012)[70] | 2009 | Guangdong | Random | 63 | 63 | 3 | | 2 | | 1 | | 1 |
|  | Li L(2014)[71] | 2009-2010 | Henan | convenience | 187 | 98 | 11 | | 5 | | 7 | | 1 |

Note : MSM: men who have sex with men； HST: Heterosexual transmission ; FPD: former plasma donors；IDU: Injecting drug users；NRTI: nucleoside reverse transcriptase inhibitor; NNRTI: non-nucleoside reverse transcriptase inhibitor; PI: protease inhibitor；TSS: Truncated sequential sampling

**Reference**

1. Guo WS, Hu QH, Zhang M, Cui WG, Li H (2006) An investigation of treatment of persons infected with HIV and drug resistance in certain areas of Henan province. Chin J AIDS STD 12: 304-306.
2. Tang H, Zhan XF, Peng GP, Chen HP, Peng TH, et al. (2007) Analyis of mutations of drug-resistant HIV-1 protease and reverse transcriptase genes in treatment-naive patients in Hubei Province. China Tropical Medicine 7: 1505-1507.
3. Cao XL, Yuan Y, Liu CH, Liu HW, Cui WG, et al. (2010) Immunology and virology and drug resistance survey among the patients infected by HIV-1 before antiviral therapy in Henan. Henan J Prev Med 21: 403-404,416.
4. Li L, Chen L, Liang S, Liu W, Li T, et al. (2013) Subtype CRF01_AE dominate the sexually transmitted human immunodeficiency virus type 1 epidemic in Guangxi, China. J Med Virol 85: 388-395.
5. Liao L, Xing H, Li X, Ruan Y, Zhang Y, et al. (2007) Genotypic analysis of the protease and reverse transcriptase of HIV type 1 isolates from recently infected injecting drug users in western China. AIDS Res Hum Retroviruses 23: 1062-1065.
6. Yu GL, LI J, Diao LM, Yan XG, Lin P, et al. (2009) Study on pol gene polymorphism and drug resistance in newly diagnosed HIV-1 infected addicts in Guangdong province. Chin J AIDS STD 15: 7-10.
7. Han XX, Zhao B, Sun F, An MM, Yin LL, et al. (2012) Primary HIV-1drug resistance among injecting drug users in Xinjiang. Chin J Public Health 28: 810-811.
8. Zhang X, Li S, Li X, Li X, Xu J, et al. (2007) Characterization of HIV-1 subtypes and viral antiretroviral drug resistance in men who have sex with men in Beijing, China. AIDS 21 Suppl 8: S59-65.
9. Zhao GL, Feng TJ, Hong FC, Wang F, Cai YM, et al. (2009) Study on drug-resistant gene mutation in HIV-1 infected MSM population in Shenzhen. Chin J AIDS STD 15: 589-591.
10. Zheng MN, Yu MH, Ning TL, Xia JH, Chen SH (2010) HIV-1 Genotype and transmitted drug reistance in men have sex with men, in Tianjin. Chin J Infect Dis 28: 303-308.
11. Yuan D, Qin GM, Xiao L, C. L, Niu AM, et al. (2011) Analysis on Primary Drug Resistance of HIV-1 Infected Men Having Sex With Men in Sichuan Province. J Pre Med Inf Mar 27: 169-172.
12. Wu YS, Zhang T, Wei FL, Li DM, Zhang WW, et al. (2011) Study on transmitted drug resistance in new infected or recently infected patients. Chinese Medical Association-Fifth national academic conferences of HIV/AIDS, viral hepatitis C and tropical diseases. Wu Han, Hubei.
13. Zhao B, Han X, Dai D, Liu J, Ding H, et al. (2011) New trends of primary drug resistance among HIV type 1-infected men who have sex with men in Liaoning Province, China. AIDS Res Hum Retroviruses 27: 1047-1053.
14. Li L, Lu X, Li H, Chen L, Wang Z, et al. (2011) High genetic diversity of HIV-1 was found in men who have sex with men in Shijiazhuang, China. Infect Genet Evol 11: 1487-1492.
15. Zhao GL, Yu W, Cai YM, Wang F, Hong FC, et al. (2012) Primary drug resistance of HIV-1 infected men who have sex with men in Shenzhen, China. Natl Med J China 92: 1165-1169.
16. Lei YH, Hu ZW, Wang H, Qin YZ, Li Y, et al. (2012) Primary drug resistance of HIV-1 in MSM population of Hefei, China. Chin J Viral Dis 2: 50-54.
17. Huang D, Li YF, Tan W, Zheng CL, Zhang Y, et al. (2013) Surveillance of primary drug resistance gene mutation for HIV infected men who have sex with men in Shenzhen in 2010. Modern Preventive Medicine 40: 521-522,525.
18. Li L, Han N, Lu J, Li T, Zhong X, et al. (2013) Genetic characterization and transmitted drug resistance of the HIV type 1 epidemic in men who have sex with men in Beijing, China. AIDS Res Hum Retroviruses 29: 633-637.
19. Yang J, Xing H, Niu J, Liao L, Ruan Y, et al. (2013) The emergence of HIV-1 primary drug resistance genotypes among treatment-naive men who have sex with men in high-prevalence areas in China. Arch Virol 158: 839-844.
20. Bu P, Xin H, Li ZJ, Sun XG, Shen LM, et al. (2013) Analysis of primary drug resistance in HIV infected men who have sex with men in Guizhou Province. Chin J AIDS STD 19: 328-330.
21. Chen M, Ma Y, Su Y, Yang L, Zhang R, et al. (2014) HIV-1 Genetic Characteristics and Transmitted Drug Resistance among Men Who Have Sex with Men in Kunming, China. PLoS One 9: e87033.
22. Liu L, Zheng GY, Wu XM, Gong Y, Liang S, et al. (2005) Analysis for Investigation of HIV Drug Resistance and Influencing Factors in Sichuan Province. J Prev Med Infor 21: 1-5.
23. Kuang JQ (2007) Genotypic HIV-1 Drug Resistance and Factors associated with the effect of Antiretroviral therapy Among Chinese HIV/AIDS Patients: Peking Union Medical College.
24. Zhong P, Pan Q, Ning Z, Xue Y, Gong J, et al. (2007) Genetic diversity and drug resistance of human immunodeficiency virus type 1 (HIV-1) strains circulating in Shanghai. AIDS Res Hum Retroviruses 23: 847-856.
25. Han X, Zhang M, Dai D, Wang Y, Zhang Z, et al. (2007) Genotypic resistance mutations to antiretroviral drugs in treatment-naive HIV/AIDS patients living in Liaoning Province, China: baseline prevalence and subtype-specific difference. AIDS Res Hum Retroviruses 23: 357-364.
26. Yao YP, Guo ZH, Yang JZ, Xu Y, Li XP, et al. (2008) Study of HIV-1 drug-resistance mutation among antiretroviral treatment-naive patients in Zhejiang province. Chinese Journal of Health Laboratory Technology 18: 29-32.
27. Chen X, Xing H, He JM, Zheng J, Zou XB, et al. (2008) Study on the threshold of HIV-1 drug resistance in Hunan province. Chin J Epidemiol 29: 787-789.
28. Hei FX, Li Y, Liao LJ, Ye JR, Chen Q, et al. (2009) The prevalence of HIV-1 drug resistant strains in Beijing in 2008. Chin J Microbiol Immunol 29: 499-502.
29. Yuan Y, Cao XL, Liu HW, Xing H, Liu CH, et al. (2009) Study on the transmission of drug resistant human immunodeficiency virus-1 in Henan province. Chin J Prev Med 43: 956-959.
30. Luo M, Liu H, Zhuang K, Liu L, Su B, et al. (2009) Prevalence of drug-resistant HIV-1 in rural areas of Hubei province in the People's Republic of China. J Acquir Immune Defic Syndr 50: 1-8.
31. Lin B (2010) Status and Influence Factors of HIV Drug Resistance In Shandong Province: Shandong University.
32. Liao L, Xing H, Shang H, Li J, Zhong P, et al. (2010) The prevalence of transmitted antiretroviral drug resistance in treatment-naive HIV-infected individuals in China. J Acquir Immune Defic Syndr 53 Suppl 1: S10-14.
33. Zhang J, Kang D, Fu J, Sun X, Lin B, et al. (2010) Surveillance of transmitted HIV type 1 drug resistance in newly diagnosed hiv type 1-infected patients in Shandong Province, China. AIDS Res Hum Retroviruses 26: 99-103.
34. Liu L, Ma JX, Liu YF, Zhang LF, Shen YZ, et al. (2011) Surveillance of subtype diversity and drug resistance among naive patients with human immunodeficiency virus type 1 in Shanghai. Journal of Microbes and Infections 6: 4-10.
35. Ma YL, Duan S, Chen M, Yao ST, Yang L, et al. (2011) Survey on HIV drug resistance transmission in Dehong Of Yunnan province in 2009. Chin J Epidemiol 32: 424-425.
36. Wu JJ, Xing H, Shen YL, Liao LJ, Su B, et al. (2011) Study on transmission of drug resistant human immunodeficiency virus-1 naive infection in Anhui Province in 2008. Chin J Dis Control Prev 15: 33-35.
37. Zhao J, Chen L, Xing H, Zhen CL, Yao JD, et al. (2011) Survey of HIV-1 drug resistance threshold and trend of transmitted drug resistance in 2008-2010,in Shenzhen, China. Chin J AIDS STD 17: 302-304.
38. Liang SJ, Xing H, Liu W, Liao LJ, Bi ZY, et al. (2011) HIV-1 drug resistance in recently infected individuals in Guangxi. Chin J AIDS STD 17: 503-505.
39. Zhang JF, Pan XH, Guo ZH, Zheng JL, Yang JZ, et al. (2012) Survey of HIV drug resistance threshold in Zhejiang province from 2009 to 2011. Chin J Prev Med 46: 519-523.
40. Xue XJ, Hong KX, Cui WG, Liu CH, Liu J, et al. (2012) The primary HIV drug resistance in partial region of Henan province. Chin J Prev Med 46: 992-994.
41. Chen M, Ma YL, Chu CX, Xing H, Xu YS, et al. (2012) Survey on the transmission of HIV drug resistance in Kunming Yunnan province in 2010. Chin J Epidemiol 33: 75-77.
42. Chen M, Wang JB, Xing H, Ma YL, Yao ST, et al. (2012) Threshold survey on HIV-1 drug resistance in Dehong of Yunnan province in 2011. Chin J Epidemiol 33: 1096-1097.
43. Xue XJ, Xing H, Liu CH, Cui WG, Tian SA, et al. (2012) Study on drug resistant mutations for HIV-1 strains in 2007-2011, in Henan Province. Chin J Prev Med 46: 659-660.
44. Wu J, Xue YL, Kang LY (2012) Molecular epidemiology and drug resistance: Survey on human immunodeficiency virus type 1 in Luwan District of Shanghai. J Diagn Concepts Pract 11: 278-282.
45. Yang HT, Xiao ZP, Huang XP, Hu HY, Xu XQ, et al. (2012) Threshold survey of transmitted HIV-1 drug resistance in Jiangsu province. Acta Universitatis Medicinalis Nanjing (Natural Science) 2012: 1-4.
46. Bu P, Xing H, Li ZJ, Liu Y (2012) A HIV drug resistance threshold survey(HIVDR-TS) in Guizhou Province in 2009. Chin J AIDS STD 18: 482-483.
47. Wang X, Yu MH, Liu ZQ, Zheng MN, Chen SH (2012) Study on pol gene polpolymorphism and drug resistance among antiretroviral treatment-naive patients in Tianjin. Chin J AIDS STD 18: 511-514.
48. Li WJ, Li H, Wang FX, Li YG (2012) Drug resistance mutations in HIV-1 strains of treatment-naive patients in Harbin, China. Chin J Viral Dis 2: 40-45.
49. Wang X, He C, Xing H, Liao L, Xu X, et al. (2012) Short communication: emerging transmitted HIV type 1 drug resistance mutations among patients prior to start of first-line antiretroviral therapy in middle and low prevalence sites in China. AIDS Res Hum Retroviruses 28: 1637-1639.
50. Chen M, Ma Y, Duan S, Xing H, Yao S, et al. (2012) Genetic diversity and drug resistance among newly diagnosed and antiretroviral treatment-naive HIV-infected individuals in western Yunnan: a hot area of viral recombination in China. BMC Infect Dis 12: 382.
51. Yang C, Liu S, Zhang T, Hou Y, Liu X, et al. (2012) Transmitted antiretroviral drug resistance and thumb subdomain polymorphisms among newly HIV type 1 diagnosed patients infected with CRF01_AE and CRF07_BC virus in Guangdong Province, China. AIDS Res Hum Retroviruses 28: 1723-1728.
52. Ye JR, Lu HY, Wang WS, Guo L, Xin RL, et al. (2012) The prevalence of drug resistance mutations among treatment-naive HIV-infected individuals in Beijing, China. AIDS Res Hum Retroviruses 28: 418-423.
53. Su Q, Liang H, Cen P, Bi Z, Zhou P (2012) HIV type 1 subtypes based on the pol gene and drug resistance mutations among antiretroviral-naive patients from Guangxi, Southern China. AIDS Res Hum Retroviruses 28: 725-728.
54. Chen M, Yang CJ, Dong LJ, Yang L, Shi YH, et al. (2013) Survey on HIV-1 drug resistance threshold in Honghe Prefecture of Yunnan province in 2011. Modern Preventive Medicine 40: 3246-3249,3255.
55. Qiu LJ, Wu SL, Liu XH, Xie MR, Yan PP, et al. (2013) Study on drug resistant mutations for HIV-1 strains in Fujian Province. Chin J AIDS STD 19: 6-9.
56. Li L, Sun G, Liang S, Li J, Li T, et al. (2013) Different Distribution of HIV-1 Subtype and Drug Resistance Were Found among Treatment Naive Individuals in Henan, Guangxi, and Yunnan Province of China. PLoS One 8: e75777.
57. Zeng P, Liu Y, He M, Gao Z, Zhou Y, et al. (2013) HIV-1 genotypic diversity and prevalence of drug resistance among treatment naive HIV-infected individuals in Chengdu of China. Virus Genes 47:408-413.
58. He JM, Xing H, Chen X, Zou XB, Peng JY, et al. (2013) Survey of HIV-1 drug resistance in Hunan Province in 2009-2012. Chin J Prev Med 47: 1065-1067.
59. Chen M, Jia MH, Su YZ, Yang CJ, Yang L, et al. (2013) Investigation on HIV-1 genotypes and drug resistance threshold in Wenshan Prefecture of Yunnan Province in 2012. Chin J Dis Control Prev 17: 1041-1045.
60. Chen M, Su YZ, Yang CJ, Dong LJ, Yang L, et al. (2013) HIV-1 genetics and drug resistance threshold survey in Lincang City of Yunnan Province in 2012. Chin J AIDS STD 19: 722-725.
61. Si XF, Huang HL, Wei M, Guan Q, Song YH, et al. (2004) Prevalence of drug resistance mutations among antiretroviral drug-naive HIV-1-infected patients in China. Chinese J Exp Chin Virol 18: 308-311.
62. He WH, Zhou PL, He HX, Zhang Y, P. HX, et al. (2006) Study on the resistance rate and influencing factors of HIV-l. Chin J Dis Control Prev 10: 259-261.
63. Li HP, Liu W, Liu HX, Liang SJ, Bao ZY, et al. (2007) Study on the antiviral therapy program among people with human immunodefjciency virus in Guangxi. Chin J Epidemiol 28: 338-342.
64. Zeng P, Wang J, Huang Y, Guo X, Li J, et al. (2012) The human immunodeficiency virus-1 genotype diversity and drug resistance mutations profile of volunteer blood donors from Chinese blood centers. Transfusion 52: 1041-1049.
65. Tu YQ, Tian F, Yao J, Sun GQ, Yang RG, et al. (2009) Genotypic drug resistance among recently HIV-1 infected persons in Henan. Chin J AIDS STD 15: 227-229,233.
66. Bi ZY (2009) Efficacy of 3TC+D4T+NVP regimen anti-HIV-1 treatment and drug resistance mutation: Guangxi Medical University.
67. Tu YQ, Wang MJ, Yao J, Zhu XM, Pan PL, et al. (2009) Human immunodeficiency virus-1 genotypic drug resistance among volunteer blood donors in Yunnan, China. Transfusion 49: 1865-1873.
68. Yin CY, Lu HZ, Huang XX, Li XO, Lou GQ, et al. (2011) HIV-1 drug-resistance mutations in treatment-naive patients in China. Chin J Clin Infect Dis 4: 201-205.
69. Qian YC, H. LY, Shen YL, Wang XH, Li Y, et al. (2012) A study of HIV-1subtype diversity and drug resistance in Hefei City. Chin J Dis Control Prev 16: 513-516.
70. Chen S, Cai W, He J, Vidal N, Lai C, et al. (2012) Molecular epidemiology of human immunodeficiency virus type 1 in Guangdong province of southern China. PLoS One 7: e48747.
71. Li L, Sun B, Zeng H, Sun Z, Sun G, et al. (2014) Relatively high prevalence of drug resistance among antiretroviral-naive patients from henan, central China. AIDS Res Hum Retroviruses 30: 160-164.
