# Supplementary material for: The Prevalence of HIV-1 Drug Resistance among Antiretroviral Treatment Naïve Individuals in Mainland China: A Meta-Analysis
Source: PLoS One. 2014 Oct 24;9(10):e110652. doi: 10.1371/journal.pone.0110652 (PMC4208788; doi:10.1371/journal.pone.0110652)
Supplement: Appendix S1 — Detailed search strategy. (DOCX) [file pone.0110652.s004.docx]

**Appendix S1 Detailed search strategy**

**Pubmed**

#1 HIV[Title/Abstract]

#2 AIDS[Title/Abstract]

#3 “acquired immunodefic*”[Title/Abstract]

#4 “acquired immune deficinc*”[Title/Abstract]

#5 OR (#1 - #4)

#6 "HIV"[Mesh])

#7 "HIV Infections"[Mesh]

#8 or (#5- #7)

#9 drug[Title/Abstract]

#10 resistance[Title/Abstract]

#11 resistant[Title/Abstract]

#12 (#9 and #10) or (#9 and #11)

#13 China [TW]

#14 #8 and #12 and #13

**China National Knowledge Infrastructure (CNKI) and Wanfang database**

#1 HIV[Subject headings]

#2 AIDS[Subject headings]

#3 acquired immunodeficiency [Subject headings]

#4 human immunodeficiency virus [Subject headings]

#5 #1 OR #2 OR #3 OR #4

#6 drug resistance [Subject headings]

#7 #5 and #6

**Chinese Biomedical Literature Database (CBM)**

#1 HIV[keywords]

#2 AIDS[keywords]

#3 acquired immunodeficiency [keywords]

#4 human immunodeficiency virus [keywords]

#5 OR(#1~#4)

#6 HIV[Title]

#7 AIDS[Title]

#8 acquired immunodeficiency [Title]

#9 human immunodeficiency virus [Title]

#10 OR(#6~#9)

#11 HIV[Abstract]

#12 AIDS[Abstract]

#13 acquired immunodeficiency [Abstract]

#14 human immunodeficiency virus [Abstract]

#15 OR(#11~#14)

#16 #5 OR #10 OR #15

#17 drug resistance(TW)

#18 #16 and #17
